# Distribution of *Anopheles *in Vietnam, with particular attention to malaria vectors of the *Anopheles minimus *complex

**DOI:** 10.1186/1475-2875-7-11

**Published:** 2008-01-11

**Authors:** Claire Garros, Cam Van Nguyen, Ho Dinh Trung, Wim Van Bortel, Marc Coosemans, Sylvie Manguin

**Affiliations:** 1 Institute of Research for Development (IRD), Centre of Biology and Management of Populations, Campus International de Baillarguet, CS 30 016, 34988 Montferrier sur Lez, France; 2 Université Catholique de Louvain, Unité d'Ecologie et de Biogéographie, Place Croix du Sud, 4-5, 1348 Louvain-La-Neuve, Belgium; 3 Institute of Geography, Vietnam Academy of Sciences and Technologies (VAST), 18 Hoang Quoc Viet, Hanoi, Vietnam; 4 National Institute of Malariology, Parasitology and Entomology (NIMPE), Luong The Vinh street, Tu Liem, Hanoi, Vietnam; 5 Prince Leopold Institute of Tropical Medicine (ITM), Department of Parasitology, Nationalestraat 155, B-2000 Antwerpen, Belgium; 6 Department of Biomedical Sciences, Faculty of Pharmaceutical, Veterinary and Biomedical Sciences, University of Antwerp, Belgium

## Abstract

**Background:**

The distribution of anopheline mosquitoes in Vietnam was examined, with a particular interest for the two sibling species of the *Anopheles minimus *complex (*Cellia*: Myzomyia), *An. minimus *and *Anopheles harrisoni*, respectively former species A and C. Because the morphological identification of both sibling species is difficult and may lead to misidentifications, accurate data on their respective distribution are missing. This is of fundamental importance since the two species seem to exhibit differential vectorial capacities for malaria transmission.

**Methods:**

Large entomological surveys based on cattle collections and molecular identifications of *An. minimus s.l. *were carried out in 23 sites throughout northern, central and south-eastern regions of Vietnam.

**Results:**

Based on previous molecular works and our data, the distribution of anopheline species and the relative densities of *An. minimus *and *An. harrisoni *were mapped. It is noteworthy that there was a high specific biodiversity at each study site. *Anopheles minimus s.l. *and *Anopheles sinensis *were the main anopheline species in the northern region, whereas *Anopheles aconitus *and *Anopheles vagus *were the most frequent ones in the central region. The southern limit of *An. harrisoni *was increased to the latitude of 11°N. Sympatry between both sibling species has been extended to new provinces.

**Conclusion:**

Malaria transmission is still high in central Vietnam and along bordering countries. Therefore, it is important to know and map the precise distribution of the main and secondary malaria vectors in Vietnam for applying efficient vector control programmes. Moreover, these maps should be regularly updated and linked to environmental characteristics relative to disease epidemiology, and environmental and climatic changes occurring in southeast Asia.

## Background

The main malaria vector *Anopheles minimus s.l. *(Myzomyia Series, Funestus Group) is composed of three sibling species, *An. minimus *(former species A), *Anopheles harrisoni *(former species C), and *An. minimus *species E [1]. *Anopheles minimus *and *An. harrisoni *can be sympatrically distributed over the southeast Asian mainland whereas species E is restricted to the Ishigaki Island in the Ryukyu Archipelago, Japan, a malaria free region [2,3]. Because the morphological identification of the two sympatric species is unreliable [4], accurate data on the distribution of *An. minimus *and *An. harrisoni *are missing [3]. Morphological misidentifications with the closely related sympatric species, such as *Anopheles aconitus*, *Anopheles pampanai *and *Anopheles varuna*, are common [5,6]. Recently, molecular identification assays have been developed which can be used to shed light on the specific distribution of each sibling species [7-10].

*Anopheles minimus s.l. *occurs in hilly, forested areas [5]. Northern Vietnam exhibits different types of landscapes ranging from the plains of the Red River to high mountains (3,143 meters). The most common larval habitats of *An. minimus s.l. *are streams or canals, with slow-running water partially shaded by grassy margins [5]. In central and south-eastern region of Vietnam, both *An. minimus s.l. *and *Anopheles dirus s.l. *occur [3,11]. The latter is the main malaria vector associated with forests or rubber plantations. Even if malaria epidemics have been hardly reported in northern Vietnam for the past 10 years, central Vietnam is still a high malaria risk area [12,13].

**Figure 1:**
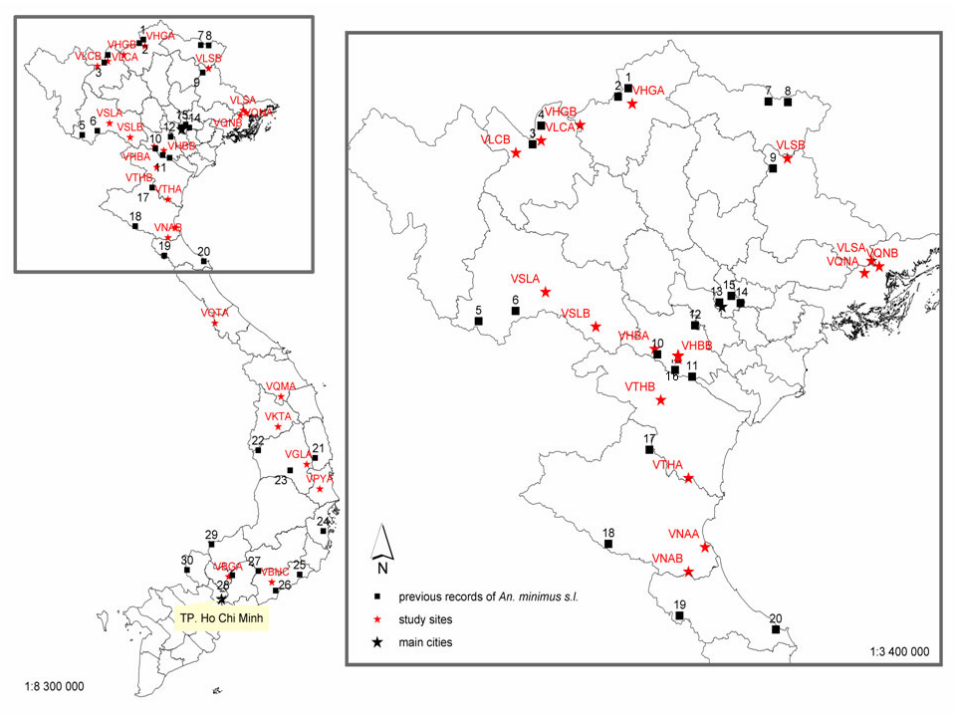
Distribution of *Anopheles minimus *and *Anopheles harrisoni *in Vietnam based on previous records and the 23 study sites (red dots). Numbers refer to Table 1. The close-up on the northern region is for a better reading.

To date, throughout Vietnam, *An. minimus *was molecularly reported in 17 provinces; and *An. harrisoni *confirmed in nine provinces (Figure 1, Table 1).

**Table 1:** Previous records of *An. minimus *and *An. harrisoni *(based on allozyme electrophoresis and molecular identifications)

Number	Locality	Date of collection	Species	Reference
1	Ha Giang Pr	March 1999	both	[10]
2			*Anopheles minimus*	
3	Lao Cai Pr	Feb. 1999	*Anopheles minimus*	
4			*Anopheles minimus*	
5	Son La Pr	Oct. 1999	both	
6			both	
7	Cao Bang Pr	Nov. 1999	both	
8			both	
9	Lang Son Pr	Apr. 2000	both	
10	Hoa Binh Pr	3–5 surveys in 1999	both	[8,10,18]
11			both	[10,29]
12			*Anopheles minimus*	
13	Hanoi Pr	10 surveys, years not given	*Anopheles minimus*	[10,18,30]
14			*Anopheles minimus*	
15			*Anopheles minimus*	
16	Thanh Hoa Pr	Nov. 1999	both	[10]
17	Nghe An Pr	Nov. 1999	*Anopheles minimus*	
18			both	
19	Ha Tinh Pr	Nov. 1999	both	
20			*Anopheles minimus*	
21	Binh Dinh Pr	Dried sample	*Anopheles minimus*	
22	Gia Lai Pr	June 1999	*Anopheles minimus*	
23			*Anopheles minimus*	
24	Khanh Hoa Pr	2001	both	[9,30]
25	Binh Thuan Pr	4 surveys in 1999	*Anopheles minimus*	[10,30]
26		2 surveys in 1999	No *Anopheles minimus s.l.*	
27		May 1999		
28	Dong Nai Pr	July 1999	*Anopheles minimus*	
29	Binh Phuoc Pr	3 surveys in 1999	*Anopheles minimus*	
30	Tay Ninh Pr	May 1999	*Anopheles minimus*	

Pr = province.

Since these sibling species may exhibit different vectorial capacities [14,15], it is fundamental to know the precise distribution of each species and to infer potential risk zones. Moreover, the southeast Asian region is currently under strong environmental changes that may alter with the vector distribution and malaria epidemiology. Therefore, the overall aim of this work was (1) to define the anopheline biodiversity in Vietnam; (2) to review the literature on molecular records of *An. minimus s.l*.; (3) to compile a distribution map for the two members of the *Anopheles minimus *complex, based on previous records and 23 new study sites.

## Methods

A total of 23 sites in 15 provinces were selected for mosquito collections in different geographical areas of northern, central and south-eastern Vietnam (Figure 1, red dots, Table 2). Adult mosquitoes were captured on cattle bait once, from October 2003 to November 2004, during a period ranging from 3 to 10 nights (Table 2). One to 5 cattle-sheds were sampled by two collectors from 21 h to midnight (Table 2). Details on mosquito collections are given in Table 2.

**Table 2:** Description of the 23 study sites (Pr = province). N = number of cattle-sheds sampled.

Village code*	Locality	GPS position	Altitude (meters)	Date of Collection	N	Type of landscape
VSLA	Son La Pr	21°08'58.3N, 104°07'32.0E	644	4–13 Apr. 2003	3	Uplands of Northern Vietnam (400–900 m)
VSLB		20°50'05.2N, 104°36'06.5E	799	15–22 Apr. 2003	3	
VHBA	Hoa Binh Pr	20°38'11.1N, 105°09'58.4E	418	2–9 May 2003	3	Landscape with limestone peaks
VHBB		20°32'4.5N, 105°23'17.8E	241	11–18 May 2003	3	Hilly areas (100–400 m) and valleys of Northern Vietnam, with isolated high hills
VHGA	Ha Giang Pr	22°50'11.7N, 104°56'55.4E	84	4–6 Nov. 2003	2	
VHGB		22°38'45.5N, 104°27'25.8E	689	12–19 Nov.2003	3	Uplands (400–900 m)
VQNA	Quanh Ninh Pr	21°19'12.8N, 107°09'42.3E	40	28 Apr.–3 May 2004	3	Hilly areas of Northern Vietnam (50–100 m) with isolated low hills
VQNB		21°22'39.0N, 107°17'57.4E	22	8–15 May 2004	3	
VTHA	Thanh Hoa Pr	19°28'48.0N, 105°29'11.2E	138	29 Apr.–9 May 2004	2	Hilly areas (100–400 m) and valleys of Central Vietnam, with isolated high hills
VTHB		20°10'50.7N, 105°13'09.0E	87	15–21 May 2004	2	Coastal plain (less than 100 m) and valleys of Central Vietnam, with isolated high hills
VNAA	Nghe An Pr	18°51'16.7N, 105°38'35.5E	14	3–10 May 2004	2	
VNAB		18°38'21.4N, 105°29'07.9E	67	16–21 May 2004	3	Hilly areas (100–400 m) and valleys of Central Vietnam, with isolated high hills
VLSA	Lang Son Pr	21°25'18.0N, 107°13'42.6E	121	4–10 June 2004	2	Hilly areas (100–400 m) and valleys, with isolated high hills
VLSB		22°20'32.1N, 106°25'19.6E	365	12–21 June 2004	3	
VLCA	Lao Cai Pr	22°30'08.3N, 104°05'03.3E	147	10–12 June 2004	3	
VLCB		22°23'42.2N, 103°50'35.1E	1,256	23–30 June 2004	3	Medium mountains (1,200–2,500 m)
VBHC	Binh Thuan Pr	11°05'17.6N, 107°53'43.7E	NA	24 Sep.–12 Oct. 2004	5	Hilly areas (100–400 m) and valleys of Central Vietnam, with isolated high hills
VQMA	Quang Nam Pr	15°09'14.9N, 108°06'25.5E	NA	30 Sep.–5 Oct 2004	1	
VGLA	Gia Lai Pr	13°39'59.4N, 108°42'24.1E	NA	1–19 Oct. 2004	3	Uplands (400–900 m)
VQTA	Quang Tri Pr	16°45'54.4N, 106°34'14.6E	NA	18–28 Oct. 2004	2	
VPYA	Phu Yen Pr	13°07'42.9N, 109°00'31.0E	NA	21–31 Oct. 2004	4	Hilly areas (100–400 m) and valleys of Central Vietnam, with isolated high hills
VKTA	Kon Tum Pr	14°29'27.5N, 108°02'30.3E	NA	27 Oct.–2 Nov. 2004	3	Low altitude (less than 900 m)

VBGA	Binh Duong Pr	11°12'19.5N, 106°53'39.6E	NA	12–20 Nov. 2004	1	High plains bordering the North of the Mekong delta (less than 100 m)

* Village code stands for V = Vietnam, second and third letters for initials of the province, A, B or C = village.

Adult mosquitoes were morphologically identified in the field using the standardized key for the medically important anophelines of southeast Asia [16]. Specimens of *An. minimus s.l. *were DNA-extracted [17] and identified with an allele specific-PCR (AS-PCR) assay [18]. Primers for *An. aconitus*, *An. pampanai *and *An. varuna *were used in the PCR mix to avoid misidentifications with the sympatric species, *An. minimus *and *An*. *harrisoni*. Positive controls of these five species identified with different molecular assays, were used [7].

Latitude/longitude position and altitude of the sites were measured with a Global Positioning System (GARMIN Etrex, Hampshire, UK) (Table 2). Mosquito densities (number of adult mosquitoes/cattle shed/night for each species) were incorporated into a database linked to the Geographic Information System package ArcView (ESRI, Redlands, California).

## Results and Discussion

**Figure 2:**
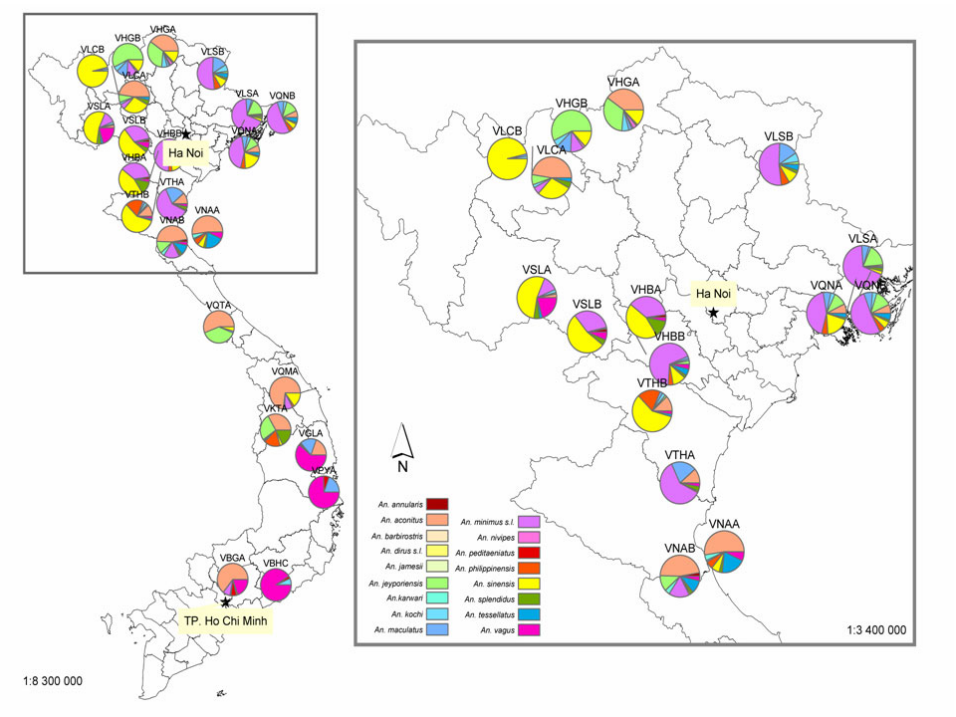
Map of the relative densities for each anopheline taxon. The close-up on northern region is for a better reading.

**Figure 3:**
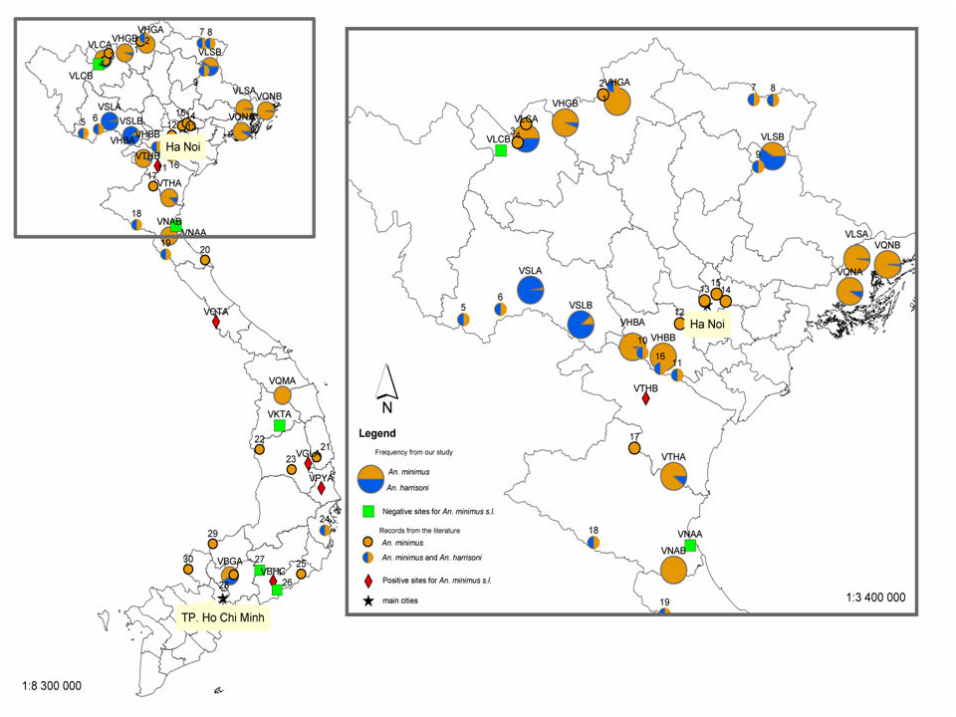
Distribution of the two sibling species of the Minimus Complex based on records obtained from this study and from the literature. Numbers refer to Table 1. The close-up on northern region is for a better reading.

This work provides updated distribution of anopheline biodiversity in Vietnam (Figure 2) and deepens and extends our knowledge on the distribution of *An. minimus, An. harrisoni *and associated anopheline species (Figures 1 and 3). Molecular records available in the literature were included to draw a complete distribution map of the Minimus complex.

The choice of collecting mosquitoes on cattle bait was intentional to have a good representation of the anopheline fauna throughout Vietnam and not to focus only on malaria vectors. In addition, studies have shown the zoophilic behaviour of known malaria vectors [15,19], which makes this collection method the most appropriate for our objective of mapping the anopheline biodiversity in Vietnam. The results illustrated and reinforced that oriental *Anopheles *are clearly opportunistic mosquitoes with trophic behaviour heterogeneities as previously found in Vietnam [15] and Thailand [20]. It is noteworthy that no individuals of *An. pampanai *and *An. varuna *were identified, although both species have already been recorded in central Vietnam (Khanh Hoa and Binh Thuan Provinces [7,8]). One assumption could be that *An. pampanai *and *An. varuna*, being so closely related to *An. aconitus *[5], may have been morphologically misidentified as *An. aconitus *[5]. The *An. aconitus *populations, identified on morphological characters only, need to be screened with the AS-PCR assay in order to avoid any potential misidentifications. If this assumption is confirmed, the density of *An. aconitus *would have been over-estimated. The results showed that *An. minimus s.l.*, main malaria vector, and secondary malaria vectors such as *An. aconitus*, are present throughout Vietnam independently of the type of landscapes, except the costal fringe where other malaria vectors are present.

Based on morphological identifications, a total of 19 anopheline species were collected and identified throughout the northern to south-eastern regions of Vietnam (Table 3) out of the 38 taxa of the Indochina fauna [12].

**Table 3:** Number of *Anopheles *specimens per species collected on cattle bait. Bottom of table: number of *An. minimus s.l. *identified by AS-PCR.

	Northern Vietnam																	Central Vietnam							
	VSLA	VSLB	VHBA	VHBB	VHGA	VHGB	VQNA	VQNB	VTHA	VTHB	VNAA	VNAB	VLSA	VLSB	VLCA	VLCB		VBHC	VQMA	VGLA	VQTA	VPYA	VKTA	VBGA		Total

*An. aconitus*	39	0	0	6	827	1	119	85	113	114	117	189	16	1	642	0		1	629	306	851	0	344	354		4,754
*An. annularis*	1	14	41	0	16	2	0	0	0	0	2	11	1	0	6	2		0	0	0	0	0	0	2		98
*An. barbirostris*	1	0	0	2	18	0	1	1	1	1	0	0	0	0	3	0		1	0	0	0	0	0	0		29
*An. campestris*	0	0	0	0	0	0	0	0	0	0	0	0	0	0	0	0		0	0	0	0	0	0	2		2
*An. dirus s.l.*	0	0	0	0	0	0	0	0	0	0	0	0	0	0	0	0		0	0	4	0	1	0	0		5
*An. jamesii*	0	0	0	0	0	0	0	0	0	0	0	0	0	0	0	0		0	0	2	0	0	0	5		7
*An. jeyporiensis*	15	4	8	37	689	565	174	167	6	14	2	5	219	17	107	0		0	2	2	578	0	277	0		2,888
*An. karwari*	0	0	0	0	1	0	0	0	0	0	14	16	0	0	0	0		0	0	0	0	0	12	0		43
*An. kochi*	33	0	5	20	19	50	55	45	0	26	0	0	20	53	32	22		55	5	5	0	1	6	2		454
*An. maculatus s.l.*	11	6	8	29	53	114	101	87	203	22	2	0	75	115	9	20		1	12	273	27	12	14	3		1,197
*An. minimus s.l.*	169	178	697	940	25	106	702	641	595	3	0	68	828	400	74	0		2	72	5	6	1	0	42		5,554
*An. nivipes*	3	0	0	0	64	19	0	0	0	0	0	0	0	0	0	0		1	0	2	0	0	3	6		98
*An. peditaenitus*	0	0	0	0	2	0	0	0	0	0	0	0	0	0	0	0		21	0	0	0	7	0	32		62
*An. philippinensis*	0	0	2	16	32	7	82	57	2	177	8	0	12	51	6	3		4	19	18	11	1	178	9		695
*An. sinensis*	742	298	805	151	260	128	305	72	11	554	10	5	34	68	386	1051		3	129	18	66	0	28	4		5,128
*An. splendidus*	62	24	87	30	5	2	25	21	36	3	0	10	31	18	66	0		0	1	0	12	0	183	0		616
*An. tessellatus*	33	1	1	71	20	0	57	64	3	18	40	41	15	35	44	5		0	0	0	0	0	0	3		451
*An. vagus*	282	35	57	54	5	3	0	0	28	24	9	14	0	2	5	4		746	1	962	0	91	9	107		2,438

Total	1,391	560	1,711	1,356	2,036	997	1,621	1,240	998	956	204	359	1,251	760	1,380	1,107		835	870	1,597	1,551	114	1,054	571		24,519

Number of An. minimus s.l. DNA-extracted	33	35	35	35	10	35	48	90	90	NA	0	44	90	96	30	0		NA	30	NA	NA	NA	0	5		706
*An. minimus*	1	4	33	35	10	33	44	88	80	NA	0	44	88	39	16	0		NA	30	NA	NA	NA	0	3		548
*An. harrisoni*	32	31	2	0	0	2	4	2	10	NA	0	0	2	57	14	0		NA	0	NA	NA	NA	0	2		158

(NA = not available)

A total of 706 *An. minimus s.l. *were DNA-extracted and identified as *An. minimus *or *An. harrisoni *for all the sites, except five sites with samples not available and three sites with no *An. minimus s.l. *populations (Table 3). Only two misidentifications in the *An. minimus *samples (two *An. minimus s.l *instead of two *An. aconitus*) were noticed for the VSLA site (northern region). No sites with *An. harrisoni *only were found neither for the northern region nor the central one (Figure 3). Interestingly enough, sites where *An. harrisoni *was dominant, *An. minimus *was often rare. This is the case in two sites in the Son La Province (northern region). This may reflect a competition between these two sibling species which needs to be further investigated. Molecular species identifications were intentionally not carried out for the Dirus and Maculatus complexes, because the Minimus complex was targeted. Recently, Obsomer *et al.*[11] published an updated review and clarified the distribution of the Dirus complex in southeast Asia.

### Northern Vietnam

*Anopheles minimus s.l. *and *An. sinensis *were the main species collected on cattle over the northern region, followed by *An. aconitus *and *An. jeyporiensis *(Figure 2). Two sites were negative for *An. minimus s.l. *(VNAA and VLCB) and one site (VTHB) presented a very low density (Table 3, Figure 2).

The VNAA village is surrounded by pine plantations. The overall number of anophelines collected was low (204 specimens during six nights) and *An. minimus s.l. *was not present during the time of our survey. The absence of *An. minimus s.l. *in the VLCB site is likely explained by the high altitude (1,256 m). Actually in Vietnam, *An. minimus s.l. *is commonly found at an altitude ranging from 200 to 800 meters and becomes quite rare at altitudes above 1,500 meters [5,21]. The low density of *An. minimus s.l. *in the VTHB site (only three specimens) may be due to very recent agricultural changes linked to modified irrigation systems that may have disturbed the mosquito larval habitats, phenomenon also observed in Thailand [22] and India [23]. The main species in this site were *An. sinensis *and *Anopheles philippinensis*, the latter species having much lower densities in all the other sites and is known to occur in agricultural areas [24].

Five sites out of 15 were negative for *An. harrisoni *(VHBB, VHGA, VNAA, VNAB, VLCB), whereas *An. minimus *was present in 13 out of 15 surveyed sites. *Anopheles harrisoni *was the predominant species of the complex in three sites only, two in the Son La Province (VSLA and VSLB), and one in the Lang Son Province (VLSB).

Regarding the distribution reported in previous studies in northern Vietnam (Figure 1), the results bring new insights on the distribution of the Minimus complex. For the first time, the anopheline composition of the extreme eastern area of the northern region was analysed and the sympatry of the two species has been reported in the three new study sites in Quanh Ninh (VQNA, VQNB) and Lang Son (VLSA) Provinces. More westwards, Phuc *et al.*[10] reported *An. minimus *only in their two sites in Lao Cai Province. However, one site (VLCA) was found positive for both sibling species in this province. Further south, the two sites VTHA and VNAB showed two different situations in the hilly areas of Thanh Hoa and Nghe An Provinces, respectively sympatric populations and *An. minimus *only.

In northern Vietnam, malaria is relatively well controlled but still occurs at the border of China and Laos [12]. However, the presence of main vectors, such as *An. minimus *and possibly *An. harrisoni*, represents a permanent threat for the resurgence of malaria in this region with the movement of infected human populations from endemic areas such as central Vietnam and bordering countries.

### Central and south-eastern Vietnam

*Anopheles aconitus *and *An. vagus *were the most frequent species in these regions (Table 3, Figure 2). *Anopheles dirus s.l. *was found in low densities in two sites only (VGLA and VPYA) (Table 3, Figure 2). It occurs typically in forests and forested fringes, either natural or man-made, such as rubber plantations where its contribution to malaria transmission is quite important in central Vietnam and throughout southeast Asia [11]. It is a highly anthropophilic mosquito [11] which explains the low densities collected on cattle during our surveys (Table 3).

One site (VKTA) was negative for *An. minimus s.l. *and four sites among the seven had low densities ranging between one and six specimens (Table 3, Figure 2). *Anopheles harrisoni *was recently found in Khanh Hoa Province [9] (site 24, Figure 1) and as recorded previously in three northern sites, the densities of this species are now more important than those of *An. minimus *which was reportedly the dominant species until 1999 [9]. This was the first record of this species in central Vietnam at a latitude of 12°N [9]. These results increased the southern limit of *An. harrisoni *to Binh Duong Province (VBGA), located in south-eastern Vietnam, at a latitude of 11°N, less than 100 km northwest of Ho Chi Minh City. This is a new site where *An. minimus *and *An. harrisoni *are sympatric.

*Anopheles aconitus*, the Maculatus complex and *An. vagus *were collected in high densities in central Vietnam (Table 3). *Anopheles maculatus s.l. *is thought to be one of the main vector in the Oriental region [25-27], and *An. aconitus *is considered a secondary vector in Bangladesh, India, Indonesia (Java) and Thailand [22,25,28,29].

## Conclusion

Based on cattle collections, *An. minimus s.l. *and *An. sinensis *were the main species in northern Vietnam, whereas *An. aconitus *and *An. vagus *were dominant in central Vietnam. *Anopheles minimus *and *An. harrisoni *of the Minimus complex are present over the northern, central and south-eastern Vietnam, down to latitude 11°N. Malaria transmission is still high in central Vietnam and along bordering countries. Future entomological surveys in the surrounding countries and, on a larger scale throughout southeast Asia, are required to molecularly identify the different members of the Minimus and Aconitus Subgroups to clarify the precise distributions of each member and to improve vector control strategies.

Compiling distribution maps based on large sample collections is not an academic exercise. Knowing precise and accurate geographic distributions of vector species is an important prerequisite for: (1) an adequate choice of zones where vector control actions should focus, (2) a better selection of future study sites for entomologists working on secondary or local vector species, (3) studies on anopheline biodiversity relating to environmental and climatic changes, (4) analyses of landscape-species associations, and (5) modelling malaria risk maps or comparison of predictive ecological maps with field observations.

Since malaria transmission still occurs in central Vietnam and population movement from the centre to the north are important, vector control measures must be maintained. In addition, entomological, epidemiological and climatic data need to be integrated into a Geographic Information System to follow the future trends of the disease and to assess the malaria risk zones. These maps should be regularly updated and linked to environmental characteristics of disease epidemiology relating to environmental and climatic changes occurring in southeast Asia.

## Competing interests

The author(s) declare that they have no competing interests.

## Authors' contributions

CG participated in the spatial and data collections in northern Vietnam, carried out the molecular identification of the Minimus samples, analysed and interpreted the data, and drafted the manuscript. CVN coordinated the spatial data collections in northern and central Vietnam and made substantial intellectual contributions to the paper. THD coordinated all the entomological surveys and contributed to the content of the paper. MC (coordinator of the Malvecasia project) and SM initiated the study and revised the paper critically for important intellectual content. All authors read and approved the final manuscript.
